# Targeted therapy for pediatric central nervous system tumors harboring mutagenic tropomyosin receptor kinases

**DOI:** 10.3389/fonc.2023.1235794

**Published:** 2023-12-07

**Authors:** Selene Cipri, Francesco Fabozzi, Giada Del Baldo, Giuseppe Maria Milano, Luigi Boccuto, Andrea Carai, Angela Mastronuzzi

**Affiliations:** ^1^ Department of Hematology/Oncology, Cell Therapy, Gene Therapies and Hemopoietic Transplant, Bambino Gesù Children’s Hospital, Istituti di Ricovero e Cura a Carattere Scientifico (IRCCS), Rome, Italy; ^2^ Department of Experimental Medicine, Sapienza University of Rome, Rome, Italy; ^3^ Healthcare Genetics Program, School of Nursing, College of Behavioral, Social and Health Sciences, Clemson University, Clemson, SC, United States; ^4^ Department of Neurosciences, Neurosurgery Unit, Bambino Gesù Children’s Hospital, Istituti di Ricovero e Cura a Carattere Scientifico (IRCCS), Rome, Italy

**Keywords:** pediatric tumors, central nervous system tumors, tropomyosin receptor kinases, NTRK, gene fusions, point mutations, acquired resistance, targeted therapy

## Abstract

The family of the neurotrophic tyrosine kinase receptor (*NTRK*) gene encodes for members of the tropomyosin receptor kinase (TRK) family. Rearrangements involving *NTRK1/2/3* are rare oncogenic factors reported with variable frequencies in an extensive range of cancers in pediatrics and adult populations, although they are more common in the former than in the latter. The alterations in these genes are causative of the constitutive activation of TRKs that drive carcinogenesis. In 2017, first-generation TRK inhibitor (TRKi) larotrectinib was granted accelerated approval from the FDA, having demonstrated histologic-agnostic activity against *NTRK*s fusions tumors. Since this new era has begun, resistance to first-generation TRKi has been described and has opened the development of second-generation molecules, such as selitrectinib and repotrectinib. In this review, we provide a brief overview of the studies on *NTRK* alterations found in pediatric central nervous system tumors and first and second-generation TRKi useful in clinical practice.

## 1 Introduction

Central nervous system (CNS) tumors are the commonest solid neoplasm in children aged 0-14 (1).

In CNS tumors, which commonly have no effective therapies, significant frequencies of neurotrophic tyrosine receptor kinase (*NTRK*) fusions have been revealed and their detection has become a cornerstone in the diagnostic evaluation of these cancers and treatment through specific therapies (2, 3). NTRKs are a family of tyrosine kinases receptors of neurotrophins implicated in neuronal development, among them the development of memory and the growth and function of neuronal synapses (4). The *NTRK1*/*2/3* genes produce three members of the tropomyosin receptor kinases (TRKs) called tropomyosin receptor kinases TRKA, TRKB and TRKC, respectively, and are characterized by an extracellular binding domain, a transmembrane region and an intracellular kinase domain (4, 5).

TRK is usually activated in tumors via fusions involving *NTRK1/2/3*, caused by rearrangements of chromosomes between *NTRK* genes, which include the kinase domain, with several partner genes. The fusion products are chimeras with a constitutively activated TRK, regardless of the ligand they bound (6, 7).

The rearrangement between tropomyosin 3 (*TPM3*) and *NTRK1* in colorectal cancer was the first detected *NTRK* fusion (8). Afterward, *NTRK* fusions were found with several partners in a wide diversity of cancer typologies: among the fusions involving *NTRK1* are known the fusions with ROS Proto-Oncogene 1, Receptor Tyrosine Kinase (*ROS1*) and Lamin A/C (*LMNA*), involved in spitzoid neoplasms and in soft tissue sarcomas (STS), respectively (9). The *LMNA-NTRK1* is involved also carcinoma of lung and colorectal (10). Translocated promoter region (*TPR*) with *NTRK1* was found in thyroid cancer, and sequestosome 1 (*SQSTM1*)-*NTRK1* fusion in STS and non-small cell lung cancer (NSCLC) (11–15). The fusion that involved ETS variant of transcription factor 6 (*ETV6*) and *NTRK3* was found, for example, in congenital fibrosarcoma, congenital mesoblastic nephroma, PTCs and colorectal cancer (9, 16–18). Regardless of this review, *NTRK* gene fusions occur in more than 2.5% of low-grade gliomas (LGGs) and 5.3% of high-grade gliomas (HGGs) in children (19), and contribute to defining infant-type hemispheric gliomas, a new type of HGG, in the 2021 WHO classification of CNS tumors (20).

In this review, we explain a brief overview of the studies on *NTRK* alterations found in pediatric CNS tumors and first- and second-generation TRKi targeted therapy.

## 2 NTRK fusions: from detection to treatment

### 2.1 Tropomyosin receptor kinase and cell cycle

Briefly, neurotrophin growth factors bind and activate TRKs in a specific manner: nerve growth factor neurotrophin (NGF) to TRKA; brain-derived neurotrophic factor (BDNF) and neurotrophin 4 (NT-4) that bins to TRKB; and neurotrophin 3 (NT-3) to all three TRK proteins, although it has a higher kinship for TRKC (21–26).

**Figure 1:**
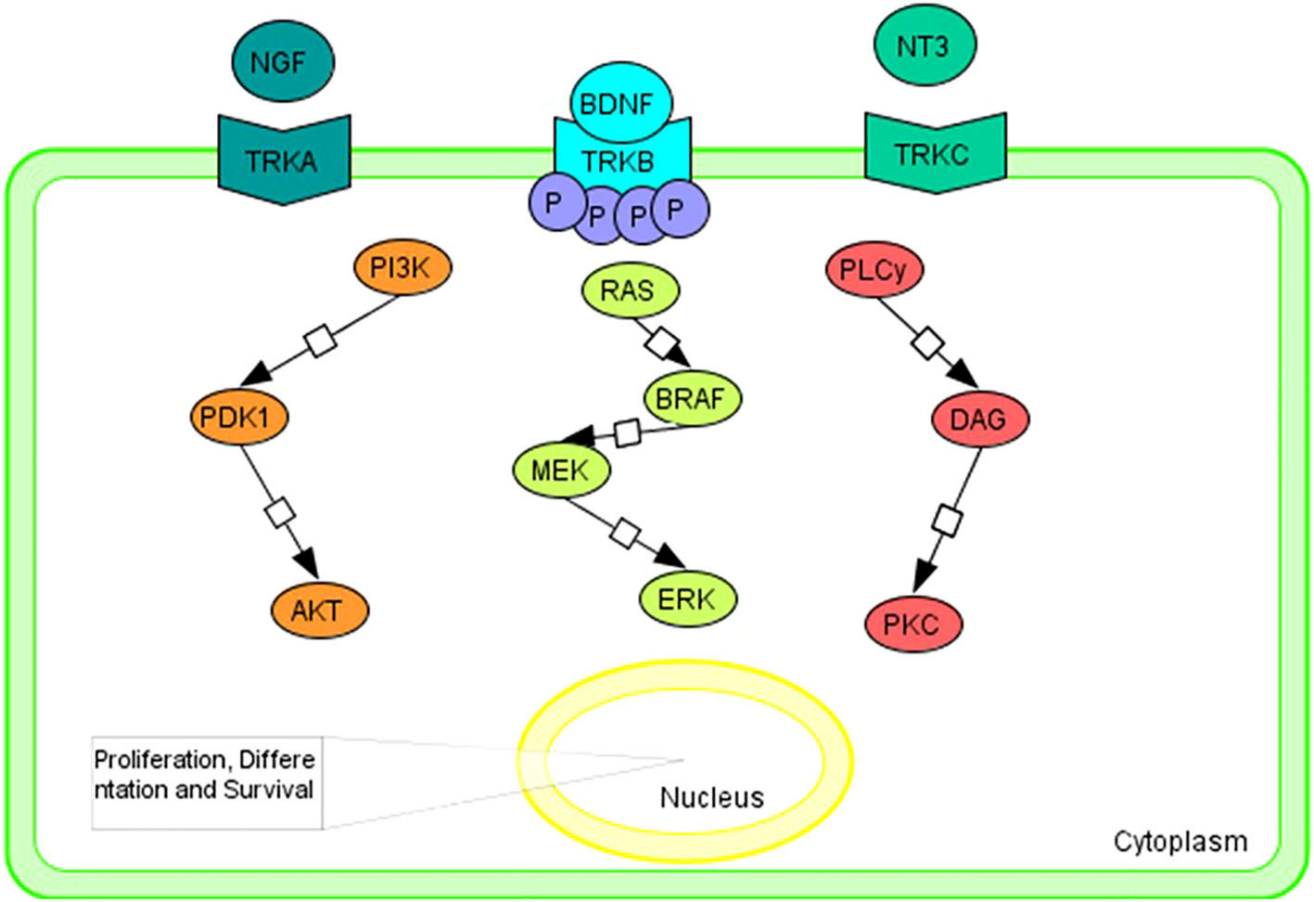
Graphical representation of the main intracellular signaling pathways associated with TRK family members. Tropomyosin receptor kinase A (TRKA); tropomyosin receptor kinase B (TRKB); tropomyosin receptor kinase C (TRKC); nerve growth factor neurotrophin (NGF); brain-derived neurotrophic factor (BDNF); neurotrophin 4 (NT-4); neurotrophin 3 (NT-3); PhosphatidylInositol 3-Kinase (PI3K); Pyruvate Dehydrogenase Kinase 1 (PDK1); AKT Serine/Threonine Kinase (AKT); B-Raf Proto-Oncogene, Serine/Threonine Kinase (BRAF); Mitogen-activated protein kinase kinase (MEK); extracellular signal-regulated kinase (ERK); Phospholipase C y (PLCy); diacylglycerol (DAG); Protein Kinase C (PKC).

The RAS/MAPK, PI3K/AKT, and PLC/PKC signaling pathway is triggered by the bond between ligand to the extracellular domain that causes the homodimerization and transactivation of TRK receptors via autophosphorylation of tyrosine residues (**Figure 1**). Activation of the above pathways promotes cell proliferation, differentiation, and survival (5, 6, 27, 28).

### 2.2 *NTRK* fusions in pediatric central nervous system tumors

In CNS tumors, significant frequencies of *NTRK* fusions have been identified and their detection has become a cornerstone in the diagnostic evaluation of these cancers (3).

Several studies including large cohorts of pediatric CNS tumors found that *NRTK1-3* alterations occur mostly in very young children and tumors localized to the hemispheric lobs (29, 30). These results converged in the 2021 WHO Classification of CNS Tumors, in which *NTRK* alterations contribute to defining novel entities among both HGGs and LGGs in children, namely infant-type hemispheric glioma and diffuse LGG, MAPK pathway‐altered, respectively (20). Despite the high-grade histology, the first subgroup benefits from a better outcome compared to its counterpart without tyrosine kinase fusions (29, 30).

**Figure 2:**
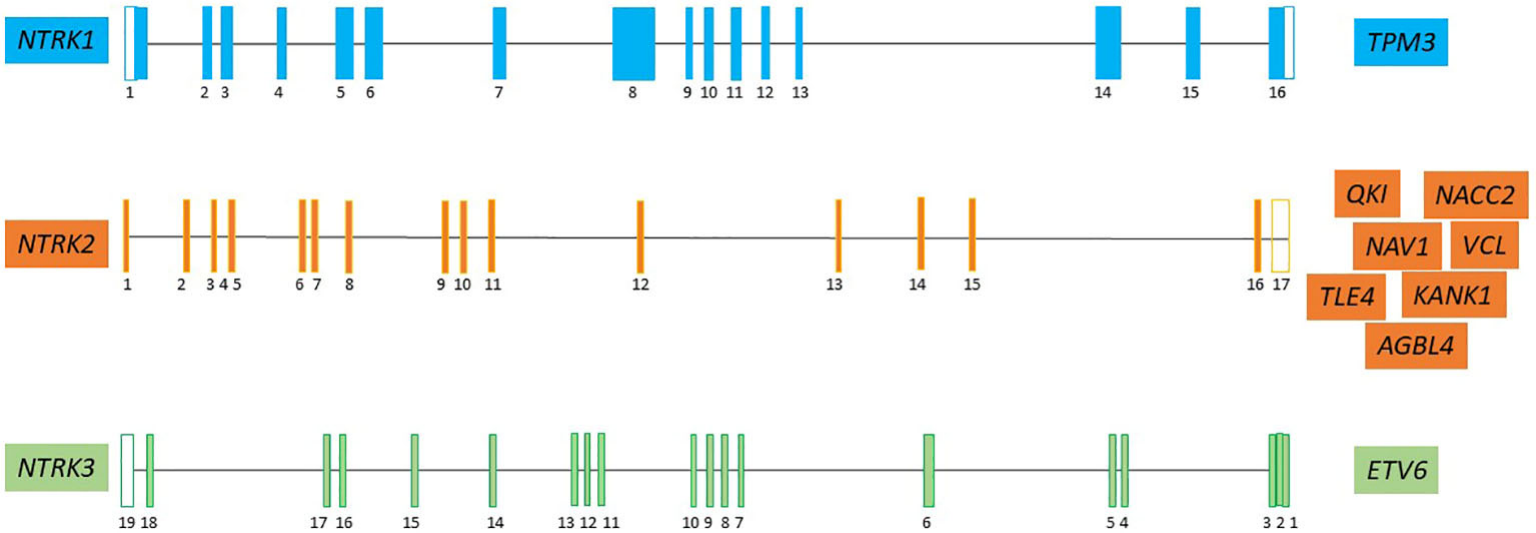
The major NTRK partner fusion genes in pediatric CNS tumor (7, 19, 28, 31–39). Neurotrophic tyrosine kinase receptor 1 (*NTRK1*), neurotrophic tyrosine kinase receptor 2 (*NTRK2*), neurotrophic tyrosine kinase receptor 3 (*NTRK3*), Tropomyosin 3 (*TPM3*); QKI, KH Domain Containing RNA Binding (*QKI*); NACC Family Member 2 (*NACC2*); Neuron Navigator 1 (*NAV1*); KN Motif And Ankyrin Repeat Domains 1 *(KANK1)*; actin-binding protein vinculin (VCL); ATP/GTP-binding protein (*AGBL4);* TLE Family Member 4, Transcriptional Corepressor (*TLE4*); ETS variant transcription factor 6 (*ETV6*).

*NTRK* fusions found in several studies are depicted in **Figure 2**.

### 2.3 NTRK inhibitors

There have been only limited *in vitro* or preclinical studies of signaling performed to illuminate the effect of TRKi on downstream cascade signaling or the time span of inhibition, but meaningful clinical responsiveness to these drugs has been shown in several types of tumors such as soft tissue sarcomas, childhood fibrosarcoma, lung cancer, colon cancer, melanoma (40–45).

First-generation TRKi were developed in 2015 that included larotrectinib and entrectinib. The recruiting clinical trials of either larotrectinib or entrectinib are listed in **Supplementary Table 1**.

Larotrectinib, developed simultaneously for pediatric and adult cancer, is the first oral treatment with a “tumor-agnostic” indication: discovered in 2015, it obtained accelerated approval from FDA in 2017. It is a small-sized competitive inhibitor of ATP and selective pan-TRK, with a 50% inhibitory concentration (IC50) of 5-11 nm *in vitro* and a specificity >100 times for TRK (46, 47). Inhibition of RAF-MEK-ERK or PI3K-AKT pathways, caused by larotrectinib, inhibits the growth of some cell lines that contained targeted *NTRK* fusions, as well *TPM3-NTRK1*, *TRIM24-NTRK2*, and *ETV6-NTRK3* (48, 49). An awesome overall response rate (ORR - the percentage of patients who experienced a complete or partial response) of 79% and a well-tolerated profile of toxicity were found in phase I/II clinical trials enrolling both adult and pediatric patients (50). In brain tumors, ORR was 30% and in particular, a retrospective study showed the results on the efficacy and safety for patients (n=33) with progressive or refractory CNS tumors enrolled in the SCOUT (NCT02637687) and NAVIGATE (NCT02576431) trials; among the 26 pediatric patients (79%), 13 pediatric HGGs and 7 pediatric LGGs were included. The observed ORR was 38% (38% in HGGs and 43% in LGGs, respectively), with three complete responses and seven partial responses. Importantly, the disease control rate at 24 weeks was 77% for pediatric HGGs and 100% for pediatric LGGs (43). In **Supplementary Table 2** are reported results on patents with CNS tumor and treated with Larotectinb.

The Food and Drug Administration approved entrectinib in August 2019 to treat adult and pediatric populations with *NTRK* fusion tumors (51).

Robinson and colleagues published the first interim results based on 29 enrolled patients, aged 5 months to 20 years. The ORR was 100% in 11 pts [(high-grade CNS tumors (n=5) and extracranial solid tumors (n=6)] (52). In 2020, an expanded cohort of 39 patients confirmed an ORR of 77%. CNS tumors were in 14 patients, of which 11 displayed *NTRK* fusions. Notably, the ORR in this subgroup reached 64% (53). Desai et al. demonstrated that entrectinib had a rapid and durable responses in pediatric patients with solid tumors harboring *NTRK1/2/3* or *ROS1* fusions (54). In **Supplementary Table 2** are reported results on patents with CNS tumor and treated with entrectinib. In addition, Liu et al. reported weight gain, dizziness and withdrawal pain in a several patients who were treated with TRKi (55).

Usually, both Larotrectinib and Entrectinib are administered until disease progression or unacceptable toxicity occurs (42, 43, 54). Treatment discontinuation is reported in extracranial tumors in which tumor size reduction has made complete resection possible; interestingly, patients who discontinued treatment following an initial response and subsequently experienced disease progression may still benefit from restart of therapy (41).

Mutations called on target and off target, respectively, on the *NTRK* gene or in genes associated with the MAPK pathway, are responsible for resistance to those drugs in several type of cancers (5, 56–59). In the *NTRK3* gene, acquired variants p.G623E and p.G623R have been identified to confer resistance to either larotrectinib or entrectinib (48, 57, 59, 60). Additionally, acquired variants p.F617L and p.G696A specifically confer resistance to larotrectinib (50, 57, 61). In the *NTRK1* gene, acquired variants p.V573M and p.G667S have been found to induce resistance to both larotrectinib and entrectinib, whereas the acquired variant p.F589L in the same gene only confers resistance to larotrectinib (50, 57, 62–64).

As a result, the need for second-generation TRKi, such as selitrectinib (loxo-195), taletrectinib (DS-6051b, AB-106), and repotrectinib (tpx-0005), has arisen (5, 30, 56). Taletrectinib works as a multi-kinase inhibitor that can overcome resistance from solvent-front replacements involving TRKA, TRKB and TRKC such as others involving ROS1 (65). Selitrectinib is a selective TRKi studied in a phase I trial involving both children and adults with tumors that have developed resistance mediated by TRK kinase mutations, in which a preliminary efficacy was found (66). Repotrectinib functions as a kinase inhibitor encoded by the *NTRK*, *ROS1*, and *ALK* genes. It effectively binds to the ATP-binding pocket of the kinase, preventing steric hindrance caused by various clinically resistant mutations (57). A clinical trial investigating its use in pediatric patients with solid tumors that include CNS neoplasms is currently ongoing (NCT04094610).

On the other hand, mutations that involved other RTKs or downstream pathway mediators can result in off-target resistance to TRKi. Specifically, MET amplification, BRAF^V600E^ mutation, or KRAS alterations have been found in patients with TRK fusion and who show a progression of the tumor during the treatment of TRKi (56). Of note, the TRKi monotherapy was not effective for resistance mediated to overcome the mutational pathway, while a dual blockade of TRK and other pathways involved in the resistance mechanism could effectively control tumor growth (67). For instance, the combination of the inhibitors of TRK and MET has been found to be effective in a patient carried a *TRK* fusion and MET amplification that drives the resistance to the TRKi alone (56).

## 3 Conclusions

Tropomyosin receptor kinase inhibitors, such as larotrectinib and entrectinib, have showed high efficacy in pediatric patients, also in CNS tumors carrying alterations in *NTRK* genes. To date, additional research is necessary to help us to understand better the mechanism of action of these drugs and to identify biomarkers that can help identify patients who will benefit most from therapy.

## Author contributions

AM, AC, and LB conceptualized the work. SC, FF, GB, and GM wrote the manuscript. AM, AC, and LB contributed to the finishing of the work and revised it critically for important intellectual content. All authors finally approved the version to be published and agreed to be accountable for all aspects of the work in ensuring that questions related to the accuracy or integrity of any part of the work are appropriately investigated and resolved. All authors contributed to the article and approved the submitted version.
