# Supplementary material for: Targeted therapy for pediatric central nervous system tumors harboring mutagenic tropomyosin receptor kinases
Source: Front Oncol. 2023 Dec 7;13:1235794. doi: 10.3389/fonc.2023.1235794 (PMC10748602; doi:10.3389/fonc.2023.1235794)
Supplement: Supplementary file 1 [file DataSheet_1.pdf]

Supplementary Material

Targeted therapy for pediatric central nervous system tumors harboring mutagenic tropomyosin receptor kinases

Selene Cipri[1], Francesco Fabozzi[1], Giada Del Baldo[1,2], Giuseppe Maria Milano[1], Luigi Boccuto[3], Andrea Carai[4], Angela Mastronuzzi[1*]

[1]Department of Hematology/Oncology, Cell Therapy, Gene Therapies and Hemopoietic Transplant, Bambino Gesù Children's Hospital, IRCCS, 00165 Rome, Italy

[2] Department of Experimental Medicine, Sapienza University of Rome, Rome, Italy

[3] Healthcare Genetics Program, School of Nursing, College of Behavioral, Social and Health Sciences, Clemson University, Clemson, SC, United States

[4]Department of Neurosciences, Neurosurgery Unit, Bambino Gesù Children's Hospital, IRCCS, 00165 Rome, Italy

*** Correspondence:** Angela Mastronuzzi
angela.mastronuzzi@opbg.net

1 Supplementary Table

Supplementary Table 1. First- and second-generation TRKi clinical trials on pediatric CNS tumor.

ID clinical trial	Phase	Status	Summary
NCT03834961	Phase 2	Active, not recruiting	This phase II trial studies the side effects and how well larotrectinib works in treating patients with previously untreated TRK fusion solid tumors and TRK fusion acute leukemia that has come back. Larotrectinib may stop the growth of cancer cells with TRK fusions by blocking the TRK enzymes needed for cell growth.
NCT04655404	Early phase 1	Recruiting	This is a pilot study that will evaluate disease status in children who have been newly diagnosed with high-grade glioma with TRK fusion. The evaluation will occur after 2 cycles of the medication (larotrectinib) have been given.<br>The study will also evaluate the safety of larotrectinib when given chemotherapy in your children; as well as the safety of larotrectinib when given post-focal radiation therapy.
NCT03213704	Phase 2	Recruiting	This phase II Pediatric MATCH trial studies how well larotrectinib works in treating patients with solid tumors, non-Hodgkin lymphoma, or histiocytic disorders with NTRK fusions that may have spread from where it first started to nearby tissue, lymph nodes, or distant parts of the body (advanced) and have come back (relapsed) or does not respond to treatment (refractory). Larotrectinib may stop the growth of cancer cells by blocking some of the enzymes needed for cell growth.
NCT03155620	Phase 2	Recruiting	This Pediatric MATCH screening and multi-sub-study phase II trial studies how well treatment that is directed by genetic testing works in pediatric patients with solid tumors, non-Hodgkin lymphomas, or histiocytic disorders that have progressed following at least one line of standard

			systemic therapy and/or for which no standard treatment exists that has been shown to prolong survival.
NCT02650401	Phase 1/2	Active, not recruiting	This is an open-label, phase I/II multicenter dose escalation study in pediatric patients with relapsed or refractory extracranial solid tumors (Phase 1), with additional expansion cohorts (Phase 2) in patients with primary brain tumors harboring NTRK1/2/3 or ROS1 gene fusions, and extracranial solid tumors harboring NTRK1/2/3 or ROS1 gene fusions.
NCT05770544	Phase 2/3	Recruiting	This trial is part of a trial program called DETERMINE. The program will also look at other anti-cancer drugs in the same way, by matching the drug to rare cancer types or ones with specific mutations.
NCT02568267	Phase 2	Active, not recruiting	This is an open-label, multicenter, global phase 2 basket study of entrectinib (RXDX-101) for the treatment of patients with solid tumors that harbor an NTRK1/2/3, ROS1, or ALK gene fusion. Patients will be assigned to different baskets according to tumor type and gene fusion.
NCT03994796	Phase 2	Recruiting	This phase II trial studies how well genetic testing works in guiding treatment for patients with solid tumors that have spread to the brain. Several genes are altered or mutated in brain metastases such as NTRK, ROS1, CDK, or PI3K. Medications that target these genes such as abemaciclib, paxalisib, and entrectinib may stop the growth of tumor cells by blocking some of the enzymes needed for cell growth. Genetic testing may help doctors tailor treatment for each mutation.
NCT03215511	Phase 1	Completed	In this trial patients with solid tumors harboring NRTK gene fusion that had progressed or were intolerant to at least one prior TRK inhibitor were enrolled in two cohorts: > 12 years and < 12 years of age, respectively.
NCT04094610	Phase 1/2	Recruiting	Phase II will evaluate the safety and tolerability at different dose levels of repotrectinib in pediatric and young adult subjects with advanced or metastatic malignancies harboring Anaplastic Lymphoma Kinase (ALK), receptor tyrosine kinase encoded by the gene ROS1 (ROS1), or neurotrophic receptor kinase genes encoding TRK kinase family (NTRK1-3) alterations to estimate the Maximum Tolerated Dose (MTD) or Maximum Administered Dose (MAD) and select the Pediatric Recommended Phase II Dose (RP2D).<br>Phase II will determine the anti-tumor activity of repotrectinib in pediatric subjects with advanced or metastatic malignancies harboring ALK, ROS1, or NTRK1-3 alterations.

2 Supplementary Table

Supplementary Table 2. Overcome of TRKi in pediatric CNS tumor with *NTRK* fusions.

Population study	Drug	Results	Reference
N= 26, including 13 pediatric HGGs and 7 pediatric LGGs were included.	Larotrectinib	ORR<br>-38% in pt with HGGs<br>-43% in pt with LGGs	43

		Three complete responses and seven partial responses.<br><br>The disease control rate at 24 weeks was 77% for pediatric HGGs and 100% for pediatric LGGs.	
N=14, including 9 primary CNS tumors (3 glioma, 2 glioblastoma, 1 astrocytoma, 3 NOS)	Larotrectinib	PR: 1 pt<br><br>SD: 7 pts<br><br>NE in 1 pt	42
N=11, including 2 pediatric HGGs with *NTRK* fusions	Larotrectinib	OS: 47 (1 pt) and 8 (1 pt)	45
N= 39 including 14 patients with CNS tumors, of which 11 displayed *NTRK* fusions.	Entrectinib	ORR = 64%.	52
N=26 including 16 patients with primary CNS tumors	Entrectinib	ORR = 50%<br><br>CR: 4 pt<br><br>PR: 4 pt<br><br>SD: 5 pt<br><br>PD: 2 pt<br><br>Missing/unavailable: 1 pt	54
